# Increased dietary availability of selenium in rainbow trout (*Oncorhynchus mykiss*) improves its plasma antioxidant capacity and resistance to infection with *Piscirickettsia salmonis*

**DOI:** 10.1186/s13567-021-00930-0

**Published:** 2021-05-01

**Authors:** Javiera Pérez-Valenzuela, Madelaine Mejías, Daniela Ortiz, Pablo Salgado, Liliana Montt, Ignacio Chávez-Báez, Francisca Vera-Tamargo, Dinka Mandakovic, Jurij Wacyk, Rodrigo Pulgar

**Affiliations:** 1 Laboratorio de Genómica y Genética de Interacciones Biológicas (LG2IB), Instituto de Nutrición y Tecnología de los Alimento, Universidad de Chile, El Líbano, Macul, 5524 Santiago, Chile; 2 Center for Research and Innovation in Aquaculture (CRIA), Universidad de Chile, Santiago, Santiago, Chile; 3 Laboratorio de Nutrición Animal (LABNA). Facultad de Ciencias Agronómicas, Producción Animal, Universidad de Chile, 11315 Santa Rosa, La Pintana Chile; 4 GEMA Center for Genomics, Ecology and Environment, Universidad Mayor, Camino La Pirámide, 5750 Huechuraba, Santiago, Chile

**Keywords:** *Piscirickettsia salmonis*, Selenium, Diet, Salmonid, Infection, Host-directed therapy

## Abstract

Salmonid Rickettsial Septicaemia (SRS), caused by *Piscirickettsia salmonis*, is the most important infectious disease in the Chilean salmon farming industry. An opportunity to control this disease is to use functional micronutrients to modulate host mechanisms of response to the infection. Since *P. salmonis* may affect the host antioxidant system in salmonids, particularly that dependent on selenium (Se), we hypothesized that fish’s dietary selenium supplementation could improve the response to the bacterial infection. To address this, we defined a non-antibiotic, non-cytotoxic concentration of selenium to evaluate its effect on the response to in vitro infections of SHK-1 cells with *P. salmonis*. The results indicated that selenium supplementation reduced the cytopathic effect, intracellular bacterial load, and cellular mortality of SHK-1 by increasing the abundance and activity of host glutathione peroxidase. We then prepared diets supplemented with selenium up to 1, 5, and 10 mg/kg to feed juvenile trout for 8 weeks. At the end of this feeding period, we obtained their blood plasma and evaluated its ability to protect SHK-1 cells from infection with *P. salmonis* in ex vivo assays. These results recapitulated the observed ability of selenium to protect against infection with *P. salmonis* by increasing the concentration of selenium and the antioxidant capacity in fish’s plasma. To the best of our knowledge, this is the first report of the protective capacity of selenium against *P. salmonis* infection in salmonids, becoming a potential effective host-directed dietary therapy for SRS and other infectious diseases in animals at a non-antibiotic concentration.

**Supplementary Information:**

The online version contains supplementary material available at 10.1186/s13567-021-00930-0.

## Introduction

Aquaculture is the third-largest productive sector in Chile, where the main species produced and exported are salmonids like Atlantic salmon (*Salmo salar*), Pacific salmon (*Oncorhynchus kisutch*), and Rainbow trout (*Oncorhynchus mykiss*). These species generated 1.6 billion dollars in 2019 only by the concept of exportation [1]. However, the industry has faced an increase in economic losses due to infectious diseases, among which Piscirickettsiosis or Salmon Rickettsial Septicemia (SRS) stands out. SRS has prevailed for more than 30 years without effective control and generating millionaire losses of around 450 million dollars per year, reaching the point of being considered a “chronic” problem for the sector [2]. This disease is caused by the bacterium *Piscirickettsia salmonis*, which can infect, replicate, and propagate in host cells’ intracellular vacuoles, its main cytopathic effect (CPE) [3].

Prophylactic strategies like vaccines used to fight SRS have been unsuccessful, so antibiotics are the most used approach. It has been described that more than 80% of the total antibiotics used in the Chilean salmon industry are against SRS since the doses tend to be higher than those administered to other bacterial diseases given the intracellular facultative nature of *P. salmonis* [4]*.* This strategy and the emergence of bacterial strains resistant to these antibiotics have severely impacted the country’s image, and the sustainability of the industry has been strongly questioned because of this [5]. Hence, new strategies not harmful to the fish or their environment are required to address this pathology.

A novel therapeutic strategy is host-directed therapy (HDT), which is based on the use of micronutrients and/or drugs that modulate host mechanisms essential for pathogen replication or persistence, therefore improving the host defense against the pathogen [6]. However, the challenge is to identify the cellular mechanisms relevant to fight the infection and then establish the micronutrients or drugs that disrupt those mechanisms.

Studies addressing salmonids’ transcriptional response to infection with *P. salmonis* suggest that the bacterium may affect the host antioxidant system, eventually causing cell death and necrosis in several fish tissues with SRS. In particular, Rise et al. in 2004 [7] described the regulation of genes involved in response to oxidative stress in head kidney and macrophages of Atlantic salmon infected with *P. salmonis*. Interestingly, the most highly up- and down-regulated antioxidants in *P. salmonis* infected macrophages (glutathione peroxidase and selenoprotein P, respectively) are functionally dependent on selenium. This observation has also been described by other authors that studied the response of other salmonids’ tissues to the infection with this bacterium [8–10]. Therefore, these studies suggest that the antioxidant host response, particularly dependent on selenium, could be relevant to fight *P. salmonis* infection.

Selenium is an essential micronutrient linked to various biological functions such as the antioxidant and anti-inflammatory response, the thyroid gland’s activity, fertility, and the response to infection [11]. Interestingly, seleno-deficient areas in China produce a human endemic cardiomyopathy (Keshan syndrome) attributed to the coxsackievirus B3 (CVB3), which mainly affects children and young people. The prevention of this disease is achieved with selenium supplementation in the diet from an early age [12]. Also, the supranutritional supplementation of selenium-yeast resulted in suppressing the progression of the HIV-1 viral load and the increase in CD4 + T cells in HIV-1 patients when their serum selenium increased significantly [13].

In fish, selenium deficiency has also been shown to correlate with increased mortality in channel catfish exposed to the intracellular bacterium *Edwardsiella ictaluri* [14]. Also, chinook salmon infected with *Renibacterium salmoninarum* significantly increased their mortality when fed with selenium-deficient diets compared to standard selenium concentration control diets [15]. These results suggest that selenium availability is relevant for an adequate response to intracellular pathogens.

Currently, Chilean salmon aquaculture uses organic selenium in the form of selenium-yeast to supplement the diets. Juvenile salmonids are being fed in a 1 mg Se/Kg concentration, representing the minimum concentration required to avoid a deficit in growth [16]. Since higher selenium supplementation levels (3.5–4.3 mg/Kg) have been shown to improve growth performance and antioxidant capacity of fish [17], we investigated if selenium supplementation at non-antibiotic nor-toxic concentrations has a protective capacity against *P. salmonis* in salmon macrophages infected in vitro and ex vivo. This investigation gives the first insights into a potential effective host-directed therapy based on supplementing a micronutrient in the diet of salmonids infected with *P. salmonis*, which could become a complementary dietary strategy to other therapeutic approaches to combat SRS.

## Materials and methods

### SHK-1 and bacterial culture conditions

The SHK-1 cell line (ECACC 97111106) was obtained from the European Collection of Authenticated Cell Cultures (ECACC). Cells were cultivated at 18 °C in Leibovitz L-15 medium (Gibco, USA) supplemented with 5% of inactivated fetal bovine serum (FBS, Gibco) and 0.1 μM of 2-mercaptoethanol (Sigma-Aldrich, Germany) in T-75 flasks (Corning, USA). *Piscirickettsia salmonis* LF-89 (ATTC VR-1361) used in this study was obtained from the American Type Culture Collection (ATCC) and was cultivated at 18 °C in solid and liquid SRS-broth media [18] with a constant 180 rpm stirring. Each subculture was confirmed as *P. salmonis* by Gram staining and RFLP assay [19]. For sodium selenite (Na_2_SeO_3_, Sigma-Aldrich) treatment, SHK-1 cells were seeded and cultured with Leibovitz L-15 medium supplemented with 5% of FBS (Gibco) without antibiotics in 24-well plates (Corning). Twenty-four hours later, cells approximately 80% confluent were supplemented with different concentrations (0; 0.5; 1.0; 1.5; 2.0; 10 and 100 µM) of Na_2_SeO_3_ to evaluate their viability by the trypan blue exclusion assay in Neubauer chambers [20]. In parallel, *P. salmonis* LF-89 was sub-cultured at 18 °C with an initial optical density (OD_620_) of 0.05 in SRS-broth media and supplemented with different concentrations of Na_2_SeO_3_ (0; 1.0; 2.0; 4.0; 6.0; 8.0 and 10 µM). To assess viability, the bacterial growth was measured by absorbance using the spectrophotometer Infinite® 200 PRO NanoQuant (Tecan Technologies, Switzerland). The viability of SHK-1 and *P. salmonis* was measured in ten biological replicates in at least two independent experiments for 12 days.

### Relative abundance and activity of glutathione peroxidase (Gpx1) in SHK-1

To evaluate the effect of a non-cytotoxic concentration of sodium selenite on the selenoprotein glutathione peroxidase (Gpx1), we compared the transcripts abundance and activity of Gpx1 between non-supplemented and supplemented SHK-1 cells with 1 μM of Na_2_SeO_3_ for 24 h. Briefly, the total RNA of 1 × 10^6^ SHK-1 cells was isolated by EZNA Total RNA kit I (OMEGA Bio-tek, USA) and quantified using a Qubit Fluorometric Quantitation System (Life Technologies, USA). According to standard procedures, we used 2 μg of total RNA as a template for reverse transcription reactions to synthesize cDNA using All-In-One 5X RT MasterMix (Amb, Canada). PCR was carried out on a real-time PCR System (Roche, Switzerland) using Brilliant II SYBR Green kit (Agilent Technologies, USA), and PCR conditions were 95 °C for 5 min followed by 94 °C for 15 s, 60 °C for 15 s and 72 °C for 20 s for a total of 35 cycles, using primers for glutathione peroxidase 1-like (XM_014133872.1) (F_gpx1: 5′-GGATGTGAATGGGAAGGATG-3′; R_gpx1: 5′-AGCGCTTGTAAGGATCTCCA-3′) and for elongation factor 1-alpha (XM_014177562.1) (F_ef-1a: 5′-CACCACCGGCAATCTGATCTACAA-3′; R_ef-1a: 5′-TCAGCAGCCTCCTTCTCGAACTTC-3′), which was selected as a normalizer gene. At least three technical replicates were performed for each quantification. To determine the activity of (Gpx1), we used the Paglia and Valentine method [21] with some modifications. Briefly, 1 × 10^6^ SHK-1 cells were lysed before enzyme assays by sonication three times for 5 s on Branson Digital Sonifier (SFX 550, Emerson, USA), followed by centrifugation at 3500 rpm, for 10 min at 4 °C to remove the debris. The lysate was resuspended with the reaction mixture and incubated for 4 min at 18 °C. After this, tert-butyl hydroperoxide was added to achieve final concentrations of 50 mM potassium phosphate buffer (pH 7.6), 0.63 mM reduced glutathione, 0.25 mM NADPH, 5 mM EDTA, 5 μg/mL Glutathione reductase and 0.1 mM tert-butyl hydroperoxide. After 1 min, the absorbance at 340 nm was measured for 4 min at 18 °C on the spectrophotometer Infinite® 200 PRO NanoQuant (Tecan Technologies). The enzyme activity (U), defined as one mole of NADPH oxidized per minute, was normalized with the total protein concentration measured using the Bradford method. The results of 32 measures in at least two independent experiments were expressed as a percentage of activity at 24 h with respect to the activity in the basal culture conditions, without supplementation with selenium.

### In vitro infection assay

In vitro infections of SHK-1 cells with *P. salmonis* were performed according to Caruffo et al. [22]. Briefly, 1 × 10^3^ SHK-1 cells were seeded on coverslips and cultured with Leibovitz’s L-15 medium supplemented with 5% of FBS (Gibco) without antibiotics in 24 wells plates (Corning). Twenty-four hours later, cells approximately 80% confluent were supplemented with Na_2_SeO_3_ 1 µM (controls with no Na_2_SeO_3_ supplementation) and inoculated with stationary phase bacteria at a multiplicity of infection (MOI) of 100 (1 cell: 100 bacteria). SHK-1 cells not inoculated with bacteria were also cultured as an infection control (*n* = 3). After 24 h, cells were washed twice with cold PBS and then incubated for 60 min with L-15 medium-plus gentamicin (100 µg/mL) to eliminate extracellular bacteria [23]. After incubation, cells were washed with PBS and incubated in an L-15 medium supplemented with or without Na_2_SeO_3_ as indicated above. To evaluate the cytopathic effect (CPE) caused by *P. salmonis*, SHK-1 cells were observed under an optical inverted phase contrast microscope AE31 (Motic, China) to follow the progression of the infection by image analysis (Moticam BTU10, Motic). Daily, for 10 days post-inoculation (dpi) with *P. salmonis*, images of cells of ten fields randomly obtained of each condition were used to count vacuoles. Cell viability was quantified using the trypan blue exclusion assay (Gibco) (*n* = 10). For bacterial quantification in infected SHK-1 cells, RNA was obtained from monolayers of infected SHK-1 cells and isolated by EZNA Total RNA kit I (OMEGA Bio-tek) and used as the template for reverse transcription reactions to synthesize cDNA using All-In-One 5X RT MasterMix (Amb), according to standard procedures. PCR conditions were 95 °C for 5 min followed by 94 °C for 15 s, 60 °C for 15 s and 72 °C for 20 s for a total of 35 cycles using the primers F_Ps16S (5′-AGGGAGACTGCCGGTGATA-3′) and R_ Ps16S (5′- ACTACGAGGCGCTTTCTCA -3′) to amplify the RNA gene of *P. salmonis* (NR_025980.1). At least five replicates were performed for each *P. salmonis* quantification.

### Immunofluorescence assay (IFAT)

For IFAT, 1 × 10^3^ SHK-1 cells were seeded on coverslips (*n* = 3), treated, and infected as was described above. Then, cells were fixed with 4% paraformaldehyde in PBS for 30 min, permeabilized with 0.1% saponin in PBS, and blocked with 0.1% saponin in PBS plus 3% BSA for 30 min. Intracellular *P. salmonis* was detected by incubation with specific antibodies anti-*P. salmonis* (ANGO, USA) at 1:200 dilution for 1 h at room temperature. After three washes with PBS, cells were incubated with secondary anti-mouse FITC-conjugated antibody (Thermo Fisher Scientific, USA) at 1:200 dilution for 1 h and with phalloidin-Alexa 636 (Thermo Fisher Scientifi) at 1:200 dilution for 15 min to detect polymerized actin; and DAPI (4′,6-diamidino-2-phenylindole, Thermo Fisher Scientific, 1:200 dilution) for 15 min to detect DNA. Preparations were observed in the Eclipse 50i epifluorescence microscope (Nikon, Japan) at 100 × magnification under oil immersion [23]. The images obtained were processed with the Fiji Image J program (GNU General Public License).

### Experimental diets

Three different diets were prepared using the commercial diet (BD) manufactured by Salmones Antártica (Puerto Montt, Chile) as the base for selenium (Se-yeast) supplementation by up to nominal final concentrations of 1, 5, and 10 mg Se per Kg of diet (SSD1, SSD5, and SSD10, respectively). This allowed us to work outside the range of selenium deficiency and toxicity reported in rainbow trout in Hilton et al. [16]; Hunt et al. [17], and Pacitti et al. [24]. Briefly, Se-yeast was homogenized manually in fish oil and incorporated into dry pellets using a laboratory vacuum coater (FoodSaver FFS003X, Oster, USA). Diets were formulated to be isoenergetic and isonitrogenous and to meet the National Research Council nutritional requirements for rainbow trout [25], where the only distinctive variable is the Se concentrations. The Se concentrations in diets were measured in triplicates by total x-ray reflection fluorescent (TXRF) in an S2 PICOFOX system (Bruker, USA) following the manufacturer’s instructions, according to Caruffo et al. [22]. Proximal composition analyses of the diet were performed at Instituto de Nutrición y Tecnología de los Alimentos (INTA, Universidad de Chile) according to the following procedures: dry matter was obtained after 24 h in an oven at 105 °C; ash by combustion at 450 °C for 16 h, protein (N*6.25) by the Kjeldahl method; fat by Soxhlet method; and gross energy by the calorific factor (4, 9 and 4 for proteins, lipids, and carbohydrates, respectively). The detail of the dietary composition is in Additional file 1.

### Experimental animals and trial conditions

Sixty juveniles rainbow trout (*O. mykiss*) were obtained from Salmones Antártica S.A (Puerto Montt, Chile). Fish were kept under a photoperiod of 14 h light and 10 h dark and acclimated for 10 days (fed with BD diet) to their experimental setup. Then, 20 fish were randomly distributed in three 200 L fiberglass tanks and fed with a daily ration of 2% of live weight (by hand-fed to visual satiation) divided into three equivalent portions (9:00 am, 1:00 pm, and 5:00 pm) with the experimental diets (SSD1, SSD5, and SSD10) for 8 weeks (60 days) at the Faculty of Agronomic Sciences Universidad de Chile. At the beginning of the study, there were no significant differences in weight between the tanks (Additional file 4). Each tank was supplied with 10 L/min of freshwater at constant temperature (15 ± 1 °C). The water quality parameters were monitored daily and kept in normal ranges for salmonids: chlorine maximum tolerable levels 0 mg/L, ammonium < 0.0125 mg/L, nitrite < 1 mg/L, nitrates 400 mg/L or more, total ammonia nitrogen < 1 mg/L and pH 6.5–7.2 according to Thorarensen and Farrell [26]. Also, oxygen was added to the system at a rate of 1 L/min whenever it was necessary to maintain saturation levels above 8 mg/L O_2_ or ≥ 90% oxygen saturation.

### Plasma biochemical profile and histopathological examination of the liver

Blood samples and weight records were obtained at 0 and 60 days after the beginning of the feeding trial. The fish’s final body weight from each tank was individually measured after anesthetized with tricaine methanesulphonate (Dolical 80%, Centrovet, Chile). Five fish were then taken from each tank for blood sampling (1 mL) from the caudal vein using 5 mL syringes with 21 G needles and collected in heparin tubes (4 mL, BD Vacutainer®, USA). The blood of each fish was then centrifuged at 4 °C at 3000 *g* for 10 min (MIKRO 200R, Hettich, Germany) to obtain approximately 300 μL of plasma that was assayed in an automatic biochemistry analyzer CM250 (Wiener Lab, Argentina) following the manufacturer’s instructions to determine the plasma biochemical parameters. Alkaline phosphatase (U/L) and aspartate aminotransferase (U/L) concentrations were determined (*n* = 5). For the histopathological examination of the liver, five fish taken from each tank were euthanized with an overdose of Dolical 80% (Centrovet) 60 days after the beginning of the feeding trial. One portion of each fish’s liver was kept in formalin 10% at pH 7 (Sigma-Aldrich) and were then rinsed with PBS and dehydrated in a graded series of ethanol, rinsed in xylene, infiltrated with paraffin, and embedded in paraffin blocks, according to Lee et al. [27]. Serial sections were prepared from each liver sample using a microtome (ModelRM2245, Leica Biosystems, Germany), placed on clean glass slides, dried at 40 °C for at least 48 h, and then stained with hematoxylin–eosin (Thermo Fisher Scientific). After staining, five pictures were taken per treatment at 10× from five fields randomly obtained of each condition, using a digital camera Moticam BTU10 (Motic) under an optical inverted phase contrast microscope AE31 (Motic).

### Selenium content and oxygen radical absorbance capacity (ORAC) in trout plasma

The quantification of Se in plasma at the end of the trial was performed by total x-ray reflection fluorescent (TXRF) in an S2 PICOFOX system (Bruker) using 20 μL of each plasma sampled following manufacturer’s instructions (*n* = 3 fish per condition). The hydrophilic antioxidant capacity of the plasma was obtained by ORAC, according to Prior et al. [28] with modifications. Briefly, a working solution at 20.34 mg/mL of 2,2′-Azobis (2-amidinopropane) dihydrochloride (AAPH) (Sigma-Aldrich) and a working solution of sodium fluorescein (Life Technologies) at 8 nM was prepared in phosphate buffer 75 mM (pH 7.4) (buffer A). 150 μL of sodium fluorescein solution was added at each experimental well, while blank wells received 25 μL of buffer A. Standards Trolox (Sigma-Aldrich) calibration curve (6.25, 12.5, 25, 50, 100 mM) were pipetted into appropriate wells and samples received 25 μL of the sample. The 96 well plate (Thermo Fisher Scientific) was then incubated for 30 min at 37 °C to equilibrate. A plate reader injector system with 5 mL of AAPH solution was used just before adding to the preincubated microplate, and reactions were initiated by adding 25 μL of AAPH solution. All ORAC analyses were performed on a synergy HTX (BioTek Instruments, USA) using an excitation wavelength of 493 nm and an emission filter of 515 nm, and ORAC values were calculated as described by Cao and Prior [28, 29], from five technical replicates.

### Ex vivo infection assay

To evaluate the effect of plasma from trout fed with different concentrations of selenium in the diets over the infection of SHK-1 cells with *P. salmonis*, we first inactivated the plasma by heat following the recommendations described by Sakai [30], making modifications such as changing the temperature to 46 °C for 15 min. Then, we determined the maximum heat-inactivated plasma concentration for supplementing the SHK-1 medium without affecting its viability or promoting the growth of *P. salmonis*. Under the cellular culture conditions described above, SHK-1 cells were supplemented with 0, 2, 4, 6, and 8% of trout plasma for 12 days (without selenium supplementation) (*n* = 3). In parallel, the culture medium of *P. salmonis* was supplemented with the same plasma concentrations to evaluate the bacterial growth for seven days. Media supplementation with 2% heat-inactivated trout plasma did not affect the viability of the cells or bacteria; hence this concentration was used in the following assays. Thus, to evaluate the effect of plasma from trout fed with the SSD1 and SSD5 diets over the in vitro infection of SHK-1 cells with *P. salmonis*, 2% of heat-inactivated FBS was supplemented with 2% of heat-inactivated trout plasma (pool of five fish from each condition) or 2% of heat-inactivated FBS (FBS-control). The in vitro infection was monitored daily, and after 10 days post-infection, cell viability was determined by trypan blue (*n* = 10). To evaluate the cytopathic effect (CPE) caused by *P. salmonis*, SHK-1 cells were stained with hematoxylin–eosin (H&E) according to Caruffo et al. [22], and observed under an optical inverted phase contrast microscope AE31 (Motic) to follow the progression of the infection by image analysis (Moticam BTU10, Motic). Six photos were taken in a random field captured at 10X. The number of total cells (nucleus per field), infected cells (cells with at least one vacuole), and vacuoles (cytopathic effect of the infection) were counted in each image for each condition. Also, the area (length*width) was measure with ImageJ (Java). At least three replicates were performed for each infection assay.

### Statistical analysis

Statistical analysis was performed using the GraphPad Prism 8.0.2 program (GraphPad Software, Inc). Differences in bacterial growth, cell viability, and relative *P. salmonis* load using different selenium concentrations and at different sampling points were analyzed using a two-way analysis of variance (ANOVA) followed by Bonferroni comparison test. Differences in bacterial growth, CPE cells/total cells, and *P. salmonis*-containing vacuoles (PCV) area obtained at a specific time point and a defined selenium concentration were analyzed using one-way ANOVA and Tukey multiple comparisons test. The results are presented as the mean ± standard deviation (SD). *P*-value < 0.05 was considered significant.

## Results

### Selenium increases the abundance and activity of Gpx1 at non-bactericidal and non-cytotoxic concentrations in SHK-1

**Figure 1:**
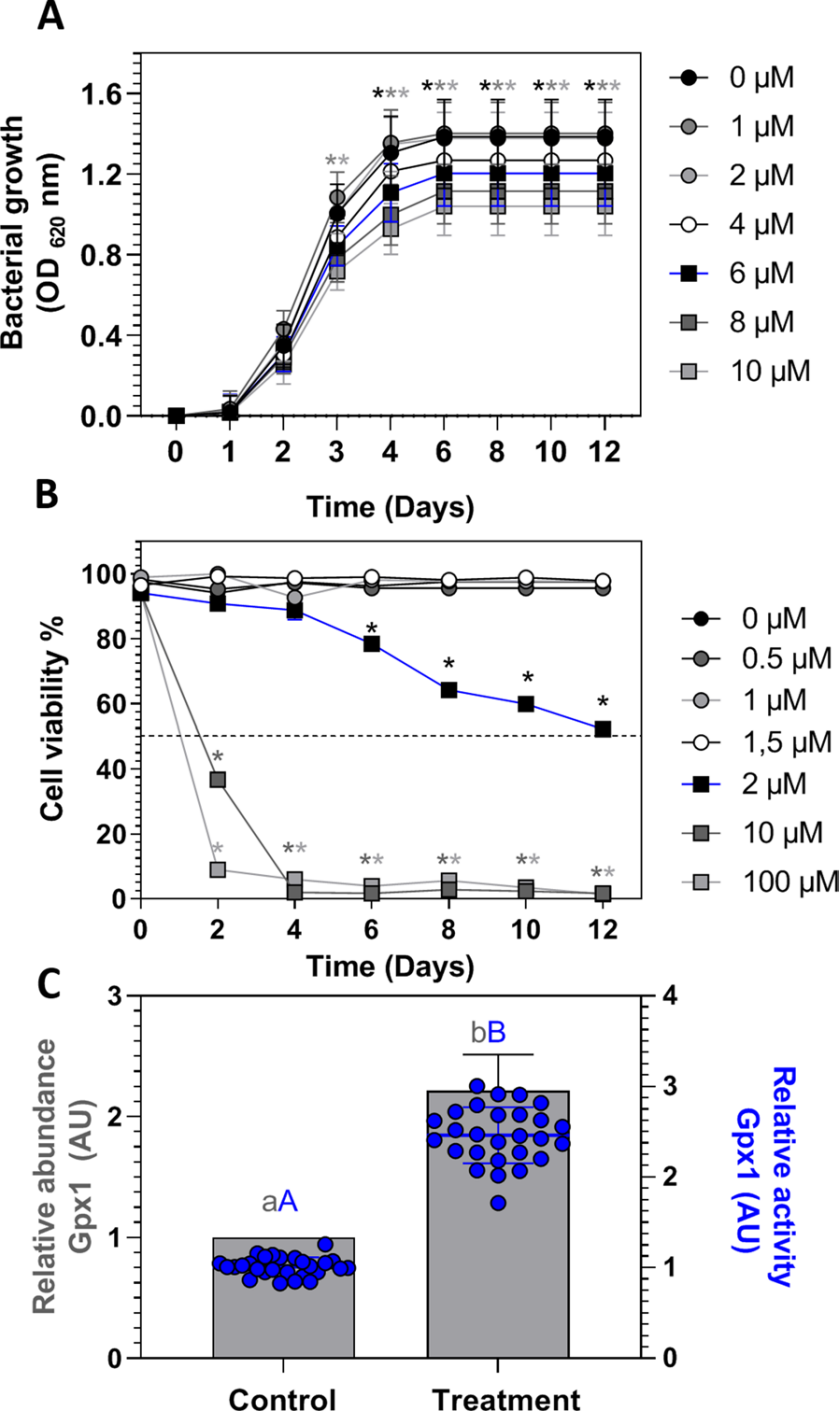
**Effect of sodium selenite on SHK-1 cell viability and** ***P. salmonis*** **growth. A** SHK-1 viability at different concentrations of sodium selenite supplemented in L-15 medium. **B** Bacterial growth of *P. salmonis* at different concentrations of sodium selenite supplemented in SRS broth. For A and B, experiments were performed for 12 days post-treatment (dpt). Each circle and square represent the mean ± SD of ten observations measured in at least two independent experiments. Bidirectional ANOVA and subsequent Bonferroni comparison test were performed with respect to the control (0 µM Na_2_SeO_3_). Colored asterisks indicate significant differences (*p*-value < 0.05) for de different condition. **C** Relative abundance (arbitrary units) and activity of glutathione peroxidase 1 (Gpx1). The glutathione peroxidase 1 experiment was carried out 24 h after treatment. The bars represent the means ± SD of the relative abundance measured in at least three independent experiments by qPCR, while the dispersion points show the glutathione peroxidase 1 activity as a result of 32 measures in at least two independent experiments. A Student *t*-test was performed for unpaired data between groups. Letters (a, A, b and B) indicate significant differences (*p*-value < 0.05).

To evaluate selenium’s effect over the host–pathogen relation and not over the host or pathogen viability, we determined the maximum concentration of sodium selenite that did not generate a cytotoxic or bactericidal effect in SHK-1 and *P. salmonis*, respectively. We grew bacteria and cells independently with sodium selenite supplements at different concentrations (0–100 μM) for 12 days. Sodium selenite showed no bactericidal effect when *P. salmonis* was grown at concentrations ranging from 0 to 4 μM. However, we observed antiproliferative effects at concentrations equal or higher to 6 μM from the third day of culture (*p*-value < 0.05; Figure 1A). Similarly, we did not record effects on the cell viability of SHK-1 at concentrations below 1.5 μM, while concentrations equal or higher to 2 μM showed reduced cell viability in a dose- and time-dependent manner (*p*-value < 0.05; Figure 1B). Therefore, the maximum concentration evaluated that did not cause a reduction in the proliferation of *P. salmonis*, or the viability of SHK-1 was 1 µM of sodium selenite; thus, this concentration was selected to perform the following in vitro experiments.

To determine the effect of sodium selenite on the mRNA abundance and activity of the selenoprotein glutathione peroxidase 1 (Gpx1) in SHK-1, we measured the abundance of the gpx1 transcript by qPCR and the enzymatic activity of Gpx1 by spectrophotometry. The results indicated that the supplementation of 1 μM of sodium selenite increased the relative abundance of gpx1 mRNA by 121.4% (2.2 AU) and the enzymatic activity of Gpx1 by 145.37% (2.5 enzyme activity) after 24 h of exposure (*p*-value < 0.05; Figure 1C) when compared to the non-supplemented control.

### Selenium reduces *Piscirickettsia salmonis* infection in SHK-1

**Figure 2:**
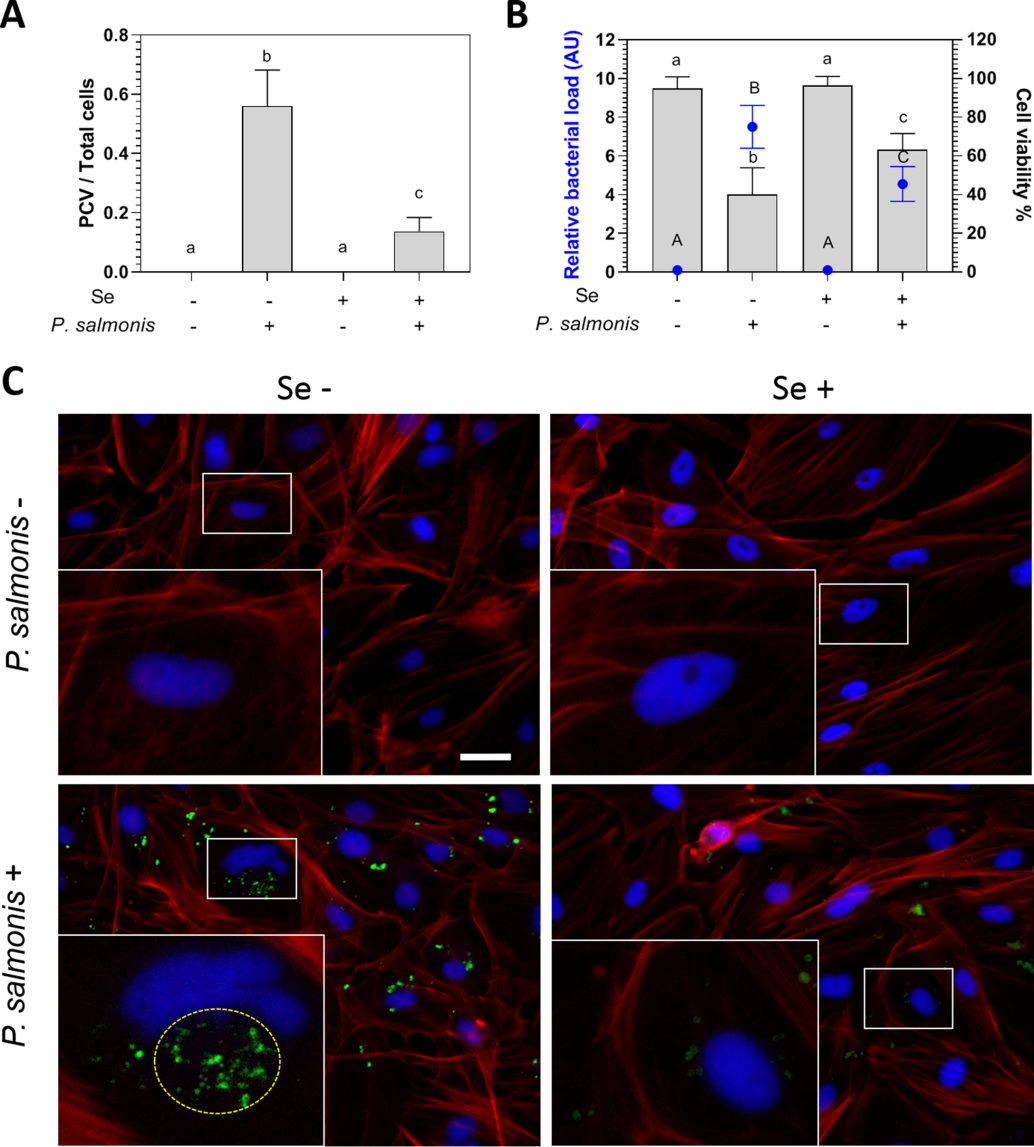
**Effect of sodium selenite in the viability of SHK-1 cells infected with** ***P. salmonis***. **A** *P. salmonis* containing vacuoles (PCVs) per total cells in SHK-1 sodium selenite treated/untreated and infected/uninfected cells at 10 days post-infection (dpi). **B** Relative *P. salmonis* load (arbitrary units, AU) (left axis) represented by blue circles and cell viability percentage (right axis) in grey bars in SHK-1 sodium selenite treated/untreated and infected/uninfected cells. **C** Representative immunofluorescence microphotographs of SHK-1 monolayer exposed (Se +) or not (Se −) to sodium selenite (1 μM). Upper panels show uninfected cells, and lower panels show *P. salmonis* infected cells at 10 dpi with sodium selenite treatment. Bar = 10 μm. For B and C, data represent mean ± SD of 10 observations measured in at least two independent experiments (*n* = 10). One-way ANOVA and Tukey multiple comparisons between all treatments were performed. Different letters represent significant differences (*p*-value < 0.05; capital letters for left axis and lowercase letters for right axis comparisons).

To investigate whether selenium exerts a protective effect on infection, we performed an in vitro infection assay with SHK-1 cells with or without sodium selenite (1 μM) and challenged with *P. salmonis.* For 10 days, we characterized the infection according to the cytopathic effect (CPE) exhibited by the infected cells, a phenomenon described by the presence of replicative vacuoles containing *P. salmonis* (PCV) in SHK-1. The results indicated that infected cells supplemented with selenium showed a significant decrease of vacuolization (*p*-value < 0.05, Figure 2A and Additional file 2) compared to infected cells not supplemented with selenium at 10 days post-infection. As we expected, this result correlated with a decrease in the bacterial load (*p*-value < 0.05, Figure 2B). Interestingly, this reduced infectious cytopathic effect in the presence of selenium was observed from 5 dpi and was maintained until the end of the trial (*p*-value < 0.05, Additional file 2). Moreover, the infected SHK-1 cells reduced their viability up to 60.1% relative to uninfected control cells in the absence of selenium; however, when the medium was supplemented with selenium, the infected cells reduced their viability only a 37.7% (*p*-value < 0.05, Figure 2B). Thus, a significant increase in cell viability in response to sodium selenite supplementation was observed in infected cells (*p*-value < 0.05). Representative images of this phenomenon are shown in the immunofluorescence of Figure 2C, in which a reduced bacterial load (green) and a decrease in PCVs (yellow dotted line) were observed in the presence of supplemented selenium (*p*-value < 0.05). The control shows how the marked PCV displaced the cell nucleus (Figure 2C), which is not observed in cells treated with selenium. Altogether, these results indicate that sodium selenite, at a non-antibiotic and non-cytotoxic concentration, reduces the cytopathic effect, bacterial load, and mortality of SHK-1 cells infected with *P. salmonis*. Besides, the results suggest that selenium exerts a protective effect by increasing the abundance and activity of glutathione peroxidase in SHK-1 infected with *P. salmonis*.

### Dietary selenium increases the plasma concentration of selenium and its antioxidant capacity in rainbow trout

**Figure 3:**
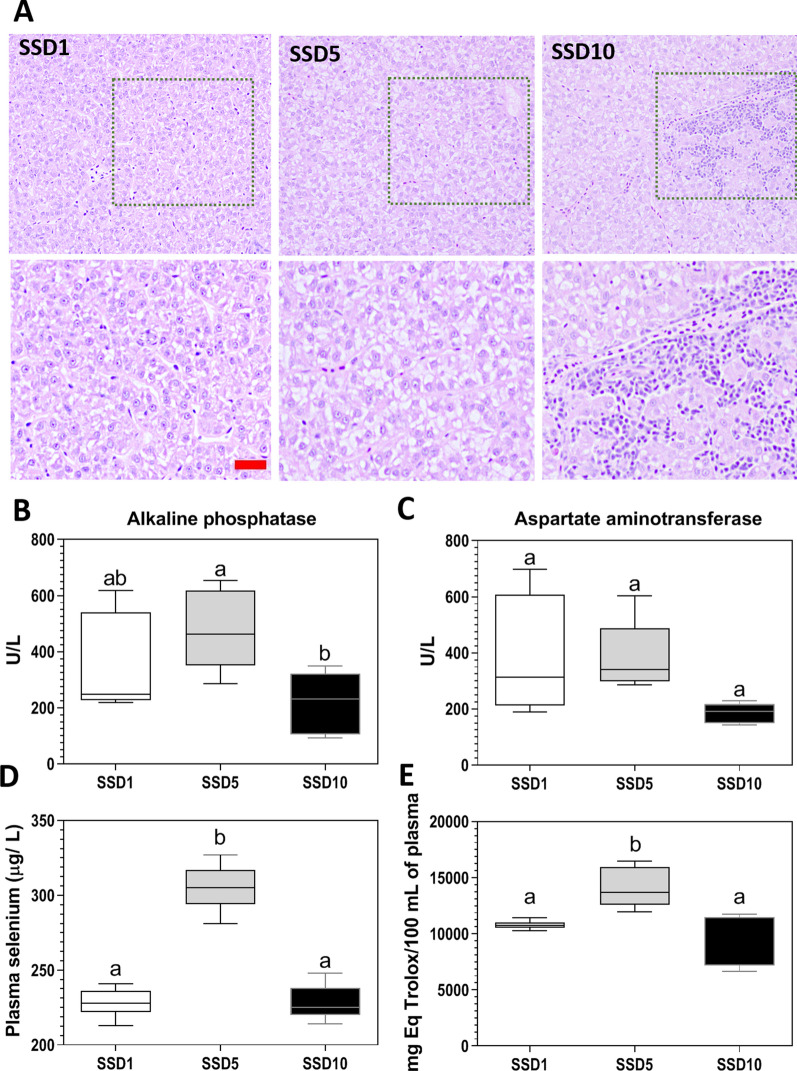
**Effect of the selenium supplemented diets on** ***O. mykiss*** **physiological traits**. **A** Representative images of livers with hematoxylin–eosin staining of fish fed with the experimental diets SSD1, SSD5, and SS10, and their respective digital zoom of 10%. Bars = 10 µm. **B** and **C** Biochemical measurements of enzymes indicative of liver damage (*n* = 5). **D** Plasma selenium concentration of *O. mykiss* fed with different concentrations of selenium in the diet measured by Total X-ray fluorescence spectroscopy (TXRF) at 60 days post-treatment (dpt) (*n* = 3). **E** Hydrophilic antioxidant capacity in plasmas from *O. mykiss* fed with different selenium concentrations in the diet measured by oxygen radical antioxidant capacity (ORAC) assay at 60 dpt (*n* = 5). Different letters represent significant differences (ANOVA *p* < 0.05, post hoc analysis Tukey).

To determine the effect of selenium on *O. mykiss* physiological parameters*,* fish were fed with selenium supplemented diets at three different concentrations: 1, 5, and 10 mg Se per Kg of diet (diets SSD1, SSD5, and SSD10, respectively) for 60 days. The livers of fish fed with SSD10 presented changes in pigmentation and friability at 30 days post-treatment (dpt), but not the livers of fish fed with diets SSD1 and SSD5 (data not shown). For this reason, at the end of the trial (60 dpt), we performed a liver histopathology assay of each condition. Representative images of the histopathology are shown in Figure 3A. The livers of fish fed with SSD1 and SSD5 had a normal phenotype, while the liver of fish fed with diet SSD10 showed signs of mononuclear cell infiltration, suggesting an inflammatory reaction associated with the consumption of a diet supplemented with 10 mg of Se per kilogram of diet. To determine if this structural observation correlated with a functional effect on hepatic enzymes, we carried out plasma determination of alkaline phosphatase (ALP) and aspartate aminotransferase (AST) activity, both markers of hepatic damage. Despite mononuclear cell infiltration, we did not observe significant differences in AST activity among the three diets. In contrast, ALP activity significantly decreased in SSP10 compared to SSP5, but not to SSP1 (*p*-value < 0.05, Figures 3B and C).

In addition, we measured the plasma concentration of selenium by Total X-ray fluorescence spectroscopy (TXRF) in fish fed with the different experimental diets. This result indicated that only diet SSD5 but not SSD10 increased the plasma selenium concentration compared to diet SSD1 (*p*-value < 0.05, Figure 3D). Interestingly, this increment of selenium correlated with a higher hydrophilic antioxidant capacity in the plasma of fish fed with diet SSD5 (*p*-value < 0.05, Figure 3E).

### High concentrations of trout plasma and selenium supplementation affects the bacterial growth and viability of SHK-1

**Figure 4:**
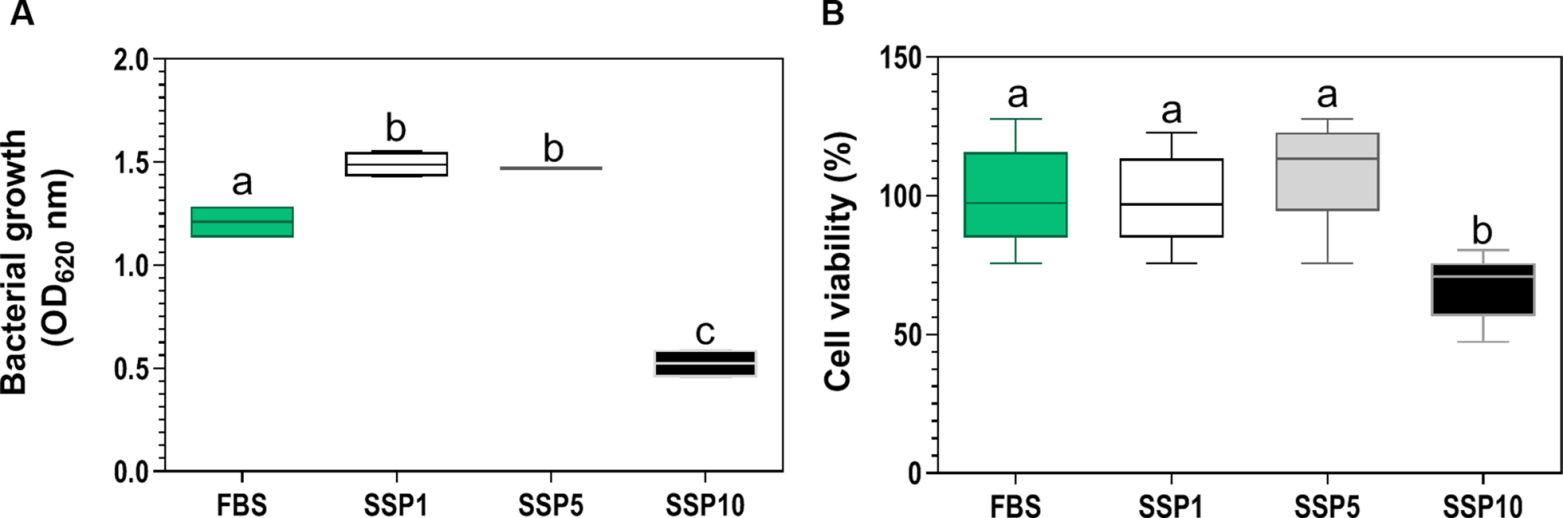
**Effect of plasma of O. mykiss fed with different selenium supplemented diets on** ***P. salmonis*** **bacterial growth and SHK-1 viability. A** Growth of *P. salmonis* in SRS-broth medium with selenium supplemented plasma (SSP) from *O. mykiss* fed with the SSD1, SSD5, or SSD10 (pool of five fish per condition) and using SRS-broth medium as a control (FBS). **B** Cell viability of SHK-1 cells in Leivobitz L-15 medium with selenium supplemented plasma (SSP) from *O. mykiss* fed with the SSD1, SSD5, or SSD10 (pool of five fish per condition) and using SRS-broth medium as a control (FBS). One-way ANOVA and Tukey multiple comparisons between all treatments were performed (*p*-value < 0.05).

To determine the maximum concentration of plasma that did not generate a bactericidal nor a cytotoxic effect in the cells of *P. salmonis* and SHK-1 cells, we first cultivated independently the bacterium and SHK-1 cells with different concentrations (0, 2, 4, 6, and 8%) of plasma from fish fed without selenium supplementation. Remarkably, bacterial growth in supplemented medium with plasma concentrations higher than 4% significantly increased *P. salmonis* growth compared to a non-supplemented medium (*p*-value < 0.05, Additional file 3). Contrarily, cellular medium supplemented with plasma concentrations equal or superior to 4% significantly decreased SHK-1 cell viability (*p*-value < 0.05, Additional file 3). Hence, we selected a 2% concentration of plasma supplementation since it did not affect the normal growth of both the bacterium and SHK-1. Then, for determining the effect of the different concentrations and antioxidant capacity of selenium in plasma, we cultured the bacterium and SHK-1 cells with a supplement of 2% of trout plasma from fish fed with diets of 1 (SSP1), 5 (SSP5), or 10 (SSP10) mg Se/ Kg and compared their growth to bacteria and SHK-1 cells cultured in media non-supplemented with fish plasma (FBS). The results indicated that despite plasma non-supplemented with selenium at 2% did not affect the bacterium and SHK-1 cells’ growth, SSP10 significantly decreased the growth and cell viability compared with the other conditions (*p*-value < 0.05, Figure 4). Interestingly, the medium supplemented with SSP1 and SSP5 did not show effects on the viability of SHK-1 compared to cells grown in FBS. Nevertheless, SSP1 and SSP5 showed an increase in the growth of *P. salmonis* compared to bacteria grown in a microbiological medium not supplemented with trout plasma (FBS). Since SSP10 displayed an antiproliferative and cytotoxic effect, it was discarded for future assays.

### Selenium supplemented trout plasma reduces *Piscirickettsia salmonis* infection in SHK-1

**Figure 5:**
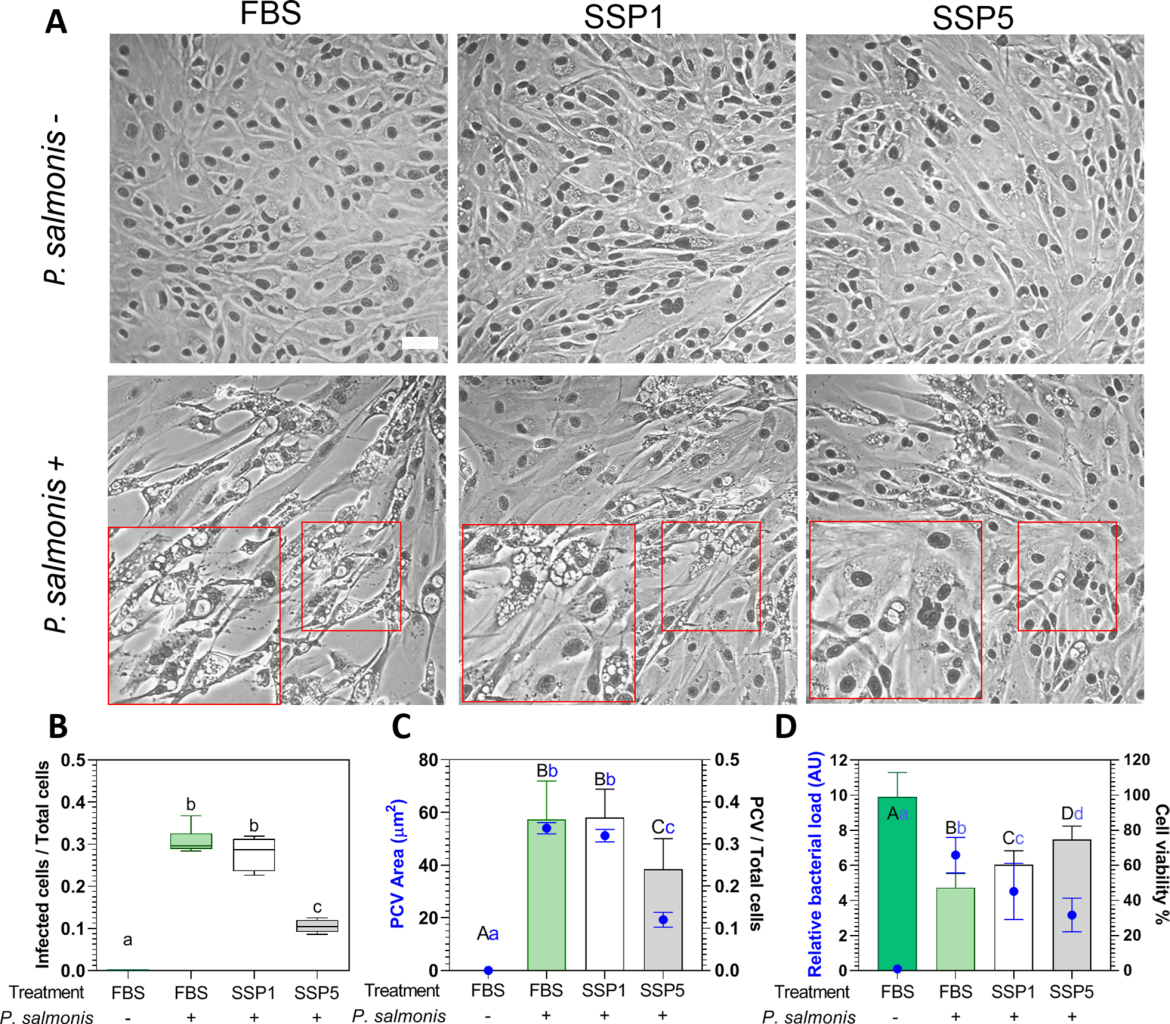
**Effect of** ***P. salmonis*** **infection in SHK-1 cells treated with plasmas obtained from *O. mykiss*** **fed with diets supplemented with different concentrations of selenium. A** Representative microphotographs of SHK-1 monolayer exposed to SSP1 or SSP5 in panoptic staining. Upper panels show uninfected cells, and lower panels show *P. salmonis* infected cells at 10 days post-infection (dpi) and selenium supplemented plasma (SSP) treatment. Red squares are indicative of *P. salmonis* containing vacuoles (PCVs), Bar = 20 μm. **B** SHK-1 cells treated and stained with hematoxylin–eosin. Six photographs were used to count infected cells with at least one per total cells on a field. **C** SHK-1 cells treated and stained with hematoxylin–eosin. Six photographs were used to count PCVs per total cells on a field represented in the right Y-axis (bars). In the left Y-axis, PCV’s area was represented (blue circles) (*n* = 6)**. D** *P. salmonis* relative load (arbitrary units (AU); left axis, blue) (*n* = 6) and cell viability measured by SHK-1 Trypan blue staining (right axis, grey) (*n* = 10) in SSP1 or SSP5 treated/untreated and infected/uninfected cells. All determinations were performed when the control was in the detachment phase of infection. One-way ANOVA and Tukey multiple comparisons between all treatments were performed. Different letters represent significant differences (*p*-value < 0.05).

Due to the high costs of performing an infection challenge in fish (in vivo), we developed an ex vivo assay to evaluate the effect of dietary selenium on fish cells in response to infections with *P. salmonis.* By doing so, we characterized the infection phenotype of *P. salmonis* infecting SHK-1 cells grown in non-supplemented (FBS) or supplemented cell culture media with 2% of SSP1 and SSP5. A significant decrease in the total number of infected cells (65.74% of reduction) and the presence of PCV (64.33% of reduction) was observed in cells treated with SSP5, but not with SSP1, when infected with *P. salmonis* and compared to non-treated cells (FBS) (*p*-value < 0.05, Figures 5A–C). Moreover, infected cells treated with SSP5 also reduced the total number of infected cells (62.09% of reduction) and the presence of PCV (62.35% of reduction) compared with cells treated with SSP1 (*p*-value < 0.05, Figures 5A–C). Interestingly, SHK-1 treated with SSP5 also significantly decreased PCV size compared to FBS and SSP1 (33.80% and 32.91% of reduction, respectively), suggesting a reduction in the infection progression. As we expected, these reduced CPEs correlated with a significant decrease in bacterial load (*p*-value < 0.05, Figure 5D). Finally, infected SHK-1 cells non treated with SSP significantly reduced their viability in comparison with non-infected SHK-1 cells (52.21% of reduction), while infected SHK-1 cells treated with SSP1 or SSP5 improved cell viability compared to infected non treated SHK-1 cells (27.85% and 57.85% of improvement, respectively) (*p*-value < 0.05, Figure 5D). Taken together, these results indicate that selenium, at a non-antibiotic and non-cytotoxic concentration, reduces the cytopathic effect, the infection progression, and the mortality of SHK-1 cells infected with *P. salmonis*.

## Discussion

Piscirickettsiosis is the most relevant infectious disease afflicting the Chilean salmon farming industry. The observed high incidence of infectious events in farms reinforces the need to generate alternative strategies to fight this disease. In this way, the use of host-directed antimicrobial drugs or micronutrients to combat intracellular bacteria like *P. salmonis* can be an opportunity to avoid the excessive use of antibiotics and their consequent effects, such as microbial resistance [6, 31, 32]. This approach allows the perturbation of host pathways used by intracellular pathogens to reproduce inside host cells, improving the defensive response by circumventing the infection and/or repressing its effects. Previous investigations on the transcriptomic response of salmonids to *P. salmonis* infection support identifying critical biological processes and pathways that are good candidates to be manipulated by this strategy [8, 9, 33], highlighting the antioxidant host metabolism.

It has been widely described that macrophages respond to the infection by producing reactive oxygen species (ROS); however, ROS is also deleterious for the host cell. Hence, an adequate antioxidant system can support this redox homeostasis based on its enzymatic machinery activity, part of which is dependent on the selenium status [34]. Selenium is an effective nutritional antioxidant that carries out biological effects by its incorporation into selenoproteins. It has been proven through various studies that selenium has a role in the immune response of different species by the regulation of the redox state of cells, influencing inflammation and immune responses [35–37]. In fish, it has been described that Chinook salmon infected with the intracellular bacterium *Renibacterium salmoninarum* have significantly increased their mortality when fed with selenium-deficient diets compared to a seleno-sufficient diet [15]. Selenium deficit has also been shown to correlate with increased mortality in channel catfish exposed to the intracellular bacterium *Edwardsiella ictaluri* [14]. Although no specific studies have been done to evaluate the effect of dietary selenium on salmonids’ response to infection with *P. salmonis*, it has been reported that several selenoproteins are consistently differentially expressed in response to the infection with this bacterium [8–10]. Therefore, we hypothesized that selenium supplementation at the host’s cellular level may affect the intracellular infection of *P. salmonis* at non-antibiotic concentrations.

Our results indicated that the classic cellular marker of selenium status, Gpx1, increased its transcript abundance and activity in the SHK-1 supplemented in vitro with selenium at non-bactericidal nor cytotoxic concentrations (*p*-value < 0.05, Figure 1). Besides, selenium’s dietary supplementation, at a non-toxic concentration, increased the selenium concentration and antioxidant capacity in trouts’ plasma (*p*-value < 0.05, Figure 3). Interestingly, direct selenium supplementation in SHK-1 cells correlated with a decreased intracellular bacterial proliferation and an increased protective capacity against *P. salmonis* infection in vitro (*p*-value < 0.05, Figure 2). This result was recapitulated when we supplied plasma from trouts fed with the SSD5 (ex vivo) to SHK-1 cells infected with *P. salmonis* at non-bactericidal nor cytotoxic concentrations (*p*-value < 0.05, Figures 4 and 5). These results emphasize the contribution of cellular models as an approach to evaluate the effects of micronutrients, such as selenium, in future applications to avoid or reduce the high costs of challenge test trials in fish.

The present report also highlights that the dietary modulation of host selenium levels must be performed with a slight adjustment, considering the risk of selenium toxicity for fundamental processes and growth traits. In this way, even though the SSD10 was under the selenium toxicity level previously described [16, 17, 24, 38], this diet generated infiltration of mononuclear cells in the liver of fish as a sign of early inflammation [39, 40] and was detrimental for the growth performance in the in vivo assay (Additional file 4). Although our objective was not to evaluate the effect of selenium on fish’s growth, our toxicity evaluation tests allowed us to observe that SSD5 significantly improved their growth compared to fish fed with SSD1 and SSD10 diets (Additional file 4), as has been recently reported by Wang et al. in 2018 [41].

Currently, Chilean aquaculture uses organic selenium in the form of selenium-yeast to supplement the salmonids’ diets at a concentration average of 1 mg Se/Kg, representing the minimum concentration required to avoid a deficit in fish’ growth [16]. However, our results suggest that concentrations up to 5 times higher in a dietary selenium supplementation (SSD5) could improve fish response to *P. salmonis* infection and its growth performance. This observation highlights that micronutrient supplementation in the fish diet should be evaluated beyond compliance with the minimum nutritional requirements and consider other aspects like the optimal response to pathogens.

To the best of our knowledge, this is the first report demonstrating the protective capacity of selenium against *P. salmonis* infection in salmonids’ macrophages. In this way, we can highlight the role that selenium plays in salmonids’ nutrition and immunological status and how an adequate supplementation of trace minerals can improve hosts’ response to intracellular pathogens, such as *P. salmonis*. Although our results show that selenium could control *P. salmonis* infection at non-antibiotic concentrations, we cannot rule out that it may exert an inhibitory activity on bacterial virulence. This aspect will be evaluated in future trials.

Finally, given the increasing prevalence of antibiotic resistance, our results collectively offer the possibility to combat SRS, and maybe other fish infections, by increasing selenium at non-antibiotic and non-toxic concentrations as a dietary adjuvant therapy to reduce the use of antibiotics.

## Supplementary Information

**Additional file 1. Ingredient formulation and nutrient composition of the basal experimental diet.** In the present study, three experimental diets were prepared by supplementing by up to 1, 5, and 10 mg/kg Se from selenium-yeast to the basal diet (BD). The resulting diets were the selenium supplemented diet 1 (SSD1), selenium supplemented diet 5 (SSD5), and selenium supplemented diet 10 (SSD10). SGR, specific growth rate. FCR, feed conversion ratio. Values are represented as mean ± SD. One-way ANOVA and Tukey multiple comparisons between all treatments were performed (*p*-value < 0.05). In each line, different letters indicate significant differences between treatments.

**Additional file 2. Effect of sodium selenite on SHK-1 cells infected with** ***P. salmonis.*** A. Representative brightfield images of SHK-1 monolayer exposed (Se +) or not (Se -) to sodium selenite (1 µM). Upper panels show uninfected cells, and lower panels show cells infected with *P. salmonis* at 10 days post-infection (dpi) and sodium selenite treatment. Bar = 20 µm. B. P. salmonis containing vacuoles (PCVs) per total cells in the field represented by boxes in SHK-1 sodium selenite treated/untreated and infected/uninfected cells at 10 dpi. The data represent mean ± SD of 10 observations measured in at least two independent experiments (*n* = 10). Bidirectional ANOVA and Bonferroni multiple comparisons between all treatments were performed (*p*-value < 0.05). Asterisks indicate significant differences.

**Additional file 3. Determination of the maximum concentration of plasma with no bactericidal nor cytotoxic effects on** ***P. salmonis*** **and SHK-1 cells.** A. Seven days post-treatment (dpt) growth of *P. salmonis* in SRS-broth medium with different wild-type plasma concentrations from *O. mykiss*. (*n* = 3). B. Supplementation of Leibovitz L-15 medium with trout plasma in SHK-1 cell (*n* = 3). For A and B, one-way ANOVA and Tukey multiple comparisons between all treatments were performed (*p*-value < 0.05). Different letters indicate significant differences.

**Additional file 4. Parameters of rainbow trout fed with selenium supplemented diets for 60 days. **SGR, specific growth rate. FCR, feed conversion ratio. Values are represented as mean ± SD (*n* = 5 fish). Different letters indicate significant differences between treatments One-way ANOVA and Tukey multiple comparisons between all treatments were performed (*p*-value < 0.05).
