# Supplementary material for: Increased dietary availability of selenium in rainbow trout (Oncorhynchus mykiss) improves its plasma antioxidant capacity and resistance to infection with Piscirickettsia salmonis
Source: Vet Res. 2021 May 1;52:64. doi: 10.1186/s13567-021-00930-0 (PMC8088010; doi:10.1186/s13567-021-00930-0)
Supplement: Supplementary file 1 — Additional file 1. Ingredient formulation and nutrient composition of the basal experimental diet. In the present study, three experimental diets were prepared by supplementing by up to 1, 5, and 10 mg/kg Se from selenium-yeast to the basal diet (BD). The resulting diets were the selenium supplemented diet 1 (SSD1), selenium supplemented diet 5 (SSD5), and selenium supplemented diet 10 (SSD10). SGR, specific growth rate. FCR, feed conversion ratio. Values are represented as mean ± SD. One-way ANOVA and Tukey multiple comparisons between all treatments were performed (p-value < 0.05). In each line, different letters indicate significant differences between treatments. [file 13567_2021_930_MOESM1_ESM.docx]

**Additional file 1 Ingredient formulation and nutrient composition of the basal experimental diet**.

|  | | | SSD1 | SSD5 | | SSD10 |
| --- | --- | --- | --- | --- | --- | --- |
| Ingredient Formulation (wet basis, g/ 100g) | | | | | |  |
|  | | | | | |  |
| Fish meal | | 67.7 | | | 67.7 | 67.7 |
| Fish oil | | 12.5 | | | 12.5 | 12.5 |
| Smolt premix* | | 7.5 | | | 7.5 | 7.5 |
| Salt (NaCl) | | 2 | | | 2 | 2 |
| Wheat gluten | | 2 | | | 2 | 2 |
| Wheat flour | | 8 | | | 8 | 8 |
| Astaxantin | | 0.5 | | | 0.5 | 0.5 |
| Se in diets (mg/Kg) |  | 1.03 ± 0.08 | | | 5.07 ± 0.11 | 10.16 ± 0.19 |
|  |  |  | | |  |  |
| Nutrient Composition (dry basis) | | | | |  |  |
| Moisture (%) | | 7.0 ± 0.2 | | | 7.0 ± 0.2 | 7.0 ± 0.2 |
| Protein (%) | | 48.6 ± 0.3 | | | 48.4 ± 0.3 | 48.7 ± 0.5 |
| Fat (%) | | 22.2 ± 1.5 | | | 22.2 ± 1.5 | 22.2 ± 1.5 |
| Ash (%) | | 10.7 ± 0.1 | | | 10.7 ± 0.2 | 10.7 ± 0.1 |
| Gross Energy (MJ/kg) | | 23.4 ± 0.3 | | | 23.4 ± 0.3 | 23.3 ± 0.3 |
|  | |  | | |  |  |

*Supplies the following per kg dry diet: KI, 1.9 mg; MnSO_4_^.^H_2_O, 75.8 mg; ZnSo_4_^.^7H_2_O, 132.0 mg; Na_2_SeO_3_, 0.88 mg; CoCl_3_^.^6H_2_O, 4.0 mg; CuSO_4_^.^H_2_O, 11.8 mg; FeSO_4_^.^H_2_O, 298.5 mg. Thiamin mononitrate, 62 mg; riboflavin, 71 mg; niacin, 294 mg; calcium pantothenate, 153 mg; pyridoxine hydrochloride, 50 mg; folic acid, 22 mg; vitamin B_12_, 0.08 mg; d-biotin, 0.8 mg; myoinositol, 176 mg; retinal acetate, 8818 IU; vitamin D_3_, 588 mg; α-tocopherol acetate, 670 mg; menadione sodium bisulfite complex, 37 mg. The Se concentrations in diets were measured in triplicates by total x-ray reflection fluorescent (TXRF) in an S2 PICOFOX system (Bruker).
