# Supplementary figures and images for: Increased dietary availability of selenium in rainbow trout (Oncorhynchus mykiss) improves its plasma antioxidant capacity and resistance to infection with Piscirickettsia salmonis
Source: Vet Res. 2021 May 1;52:64. doi: 10.1186/s13567-021-00930-0 (PMC8088010; doi:10.1186/s13567-021-00930-0)

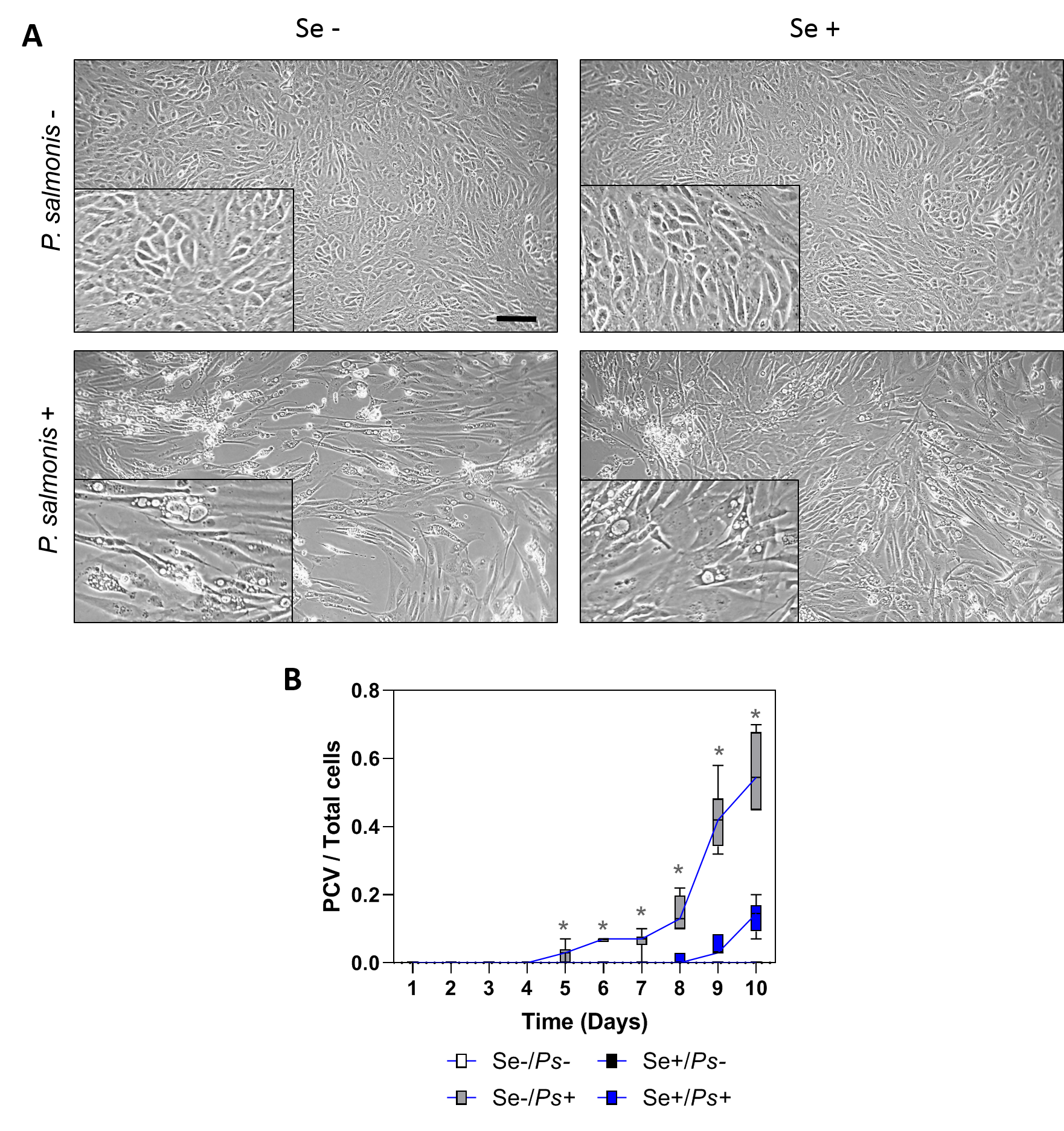

Supplement: Supplementary file 2 — Additional file 2. Effect of sodium selenite on SHK-1 cells infected with P. salmonis. A. Representative brightfield images of SHK-1 monolayer exposed (Se +) or not (Se -) to sodium selenite (1 µM). Upper panels show uninfected cells, and lower panels show cells infected with P. salmonis at 10 days post-infection (dpi) and sodium selenite treatment. Bar = 20 µm. B. P. salmonis containing vacuoles (PCVs) per total cells in the field represented by boxes in SHK-1 sodium selenite treated/untreated and infected/uninfected cells at 10 dpi. The data represent mean ± SD of 10 observations measured in at least two independent experiments (n = 10). Bidirectional ANOVA and Bonferroni multiple comparisons between all treatments were performed (p-value < 0.05). Asterisks indicate significant differences. [file 13567_2021_930_MOESM2_ESM.tif]

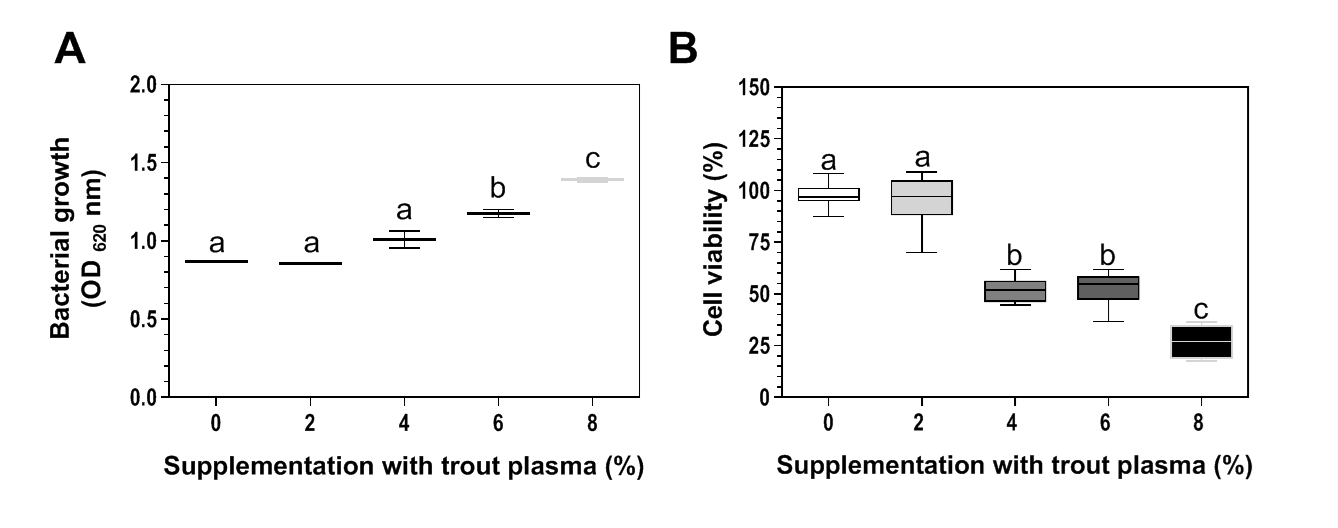

Supplement: Supplementary file 3 — Additional file 3. Determination of the maximum concentration of plasma with no bactericidal nor cytotoxic effects on P. salmonis and SHK-1 cells. A. Seven days post-treatment (dpt) growth of P. salmonis in SRS-broth medium with different wild-type plasma concentrations from O. mykiss. (n = 3). B. Supplementation of Leibovitz L-15 medium with trout plasma in SHK-1 cell (n = 3). For A and B, one-way ANOVA and Tukey multiple comparisons between all treatments were performed (p-value < 0.05). Different letters indicate significant differences. [file 13567_2021_930_MOESM3_ESM.tif]
