# Supplementary material for: Increased dietary availability of selenium in rainbow trout (Oncorhynchus mykiss) improves its plasma antioxidant capacity and resistance to infection with Piscirickettsia salmonis
Source: Vet Res. 2021 May 1;52:64. doi: 10.1186/s13567-021-00930-0 (PMC8088010; doi:10.1186/s13567-021-00930-0)
Supplement: Supplementary file 4 — Additional file 4. Parameters of rainbow trout fed with selenium supplemented diets for 60 days. SGR, specific growth rate. FCR, feed conversion ratio. Values are represented as mean ± SD (n = 5 fish). Different letters indicate significant differences between treatments One-way ANOVA and Tukey multiple comparisons between all treatments were performed (p-value < 0.05). [file 13567_2021_930_MOESM4_ESM.docx]

**Additional file 4 Parameters of rainbow trout fed with selenium supplemented diets for 8 weeks.**

|  | Diet |  |  |  |  | |
| --- | --- | --- | --- | --- | --- | --- |
|  | SSD1 | | SSD5 | | SSD10 | |
| Initial weight (g) | 238.78 | ±26.5^a^ | 241,27 | ±26.6^a^ | 242,69 | ±26.7^a^ |
| Final weight (g) | 374.8 | ±38.9^a^ | 457 | ±34.0^b^ | 382,4 | ±49.3^a^ |
| Total feed intake (g) | 4070 |  | 4076 |  | 4057 |  |
| SGR | 0.75 | ±0.2^a^ | 1,06 | ±0.2^b^ | 0,76 | ±0.3^a^ |
| FCR | 1.49 | ±0.3^a^ | 0,94 | ±0.2 ^b^ | 1,45 | ±0,3^a^ |

SGR, specific growth rate. FCR, feed conversion ratio values are represented as mean ± SD (*n* = 5 fish). In each line, different superscript letters indicate significant differences between treatments (*P* < 0.05).
